# Individual fate and gut microbiome composition in the European wild rabbit (*Oryctolagus cuniculus*)

**DOI:** 10.1038/s41598-020-80782-4

**Published:** 2021-01-12

**Authors:** Gerard Funosas, Xavier Triadó-Margarit, Francisca Castro, Rafael Villafuerte, Miguel Delibes-Mateos, Carlos Rouco, Emilio O. Casamayor

**Affiliations:** 1 Microbial Community Ecology, Centre for Advanced Studies of Blanes-Spanish Council for Research CEAB-CSIC, Accés Cala St Francesc, 14, 17300 Blanes, Spain; 2 Departamento de Didácticas Específicas, Universidad de Córdoba, Sociedad, Ecología y Gestión del Medio Ambiente, UCO-IESA, Unidad Asociada al CSIC, 14004 Córdoba, Spain; 3 Institute of Advanced Social Studies-Spanish Council for Research (IESA-CSIC), 14004 Córdoba, Spain; 4 Ecology Area, Faculty of Science, University of Cordoba, Sociedad, Ecología y Gestión del Medio Ambiente, UCO-IESA, Unidad Asociada al CSIC, 14071 Córdoba, Spain

**Keywords:** Conservation biology, Microbial ecology, Population dynamics

## Abstract

Studies connecting microbiome composition and functional performance in wildlife have received little attention and understanding their connections with wildlife physical condition are sorely needed. We studied the variation in gut microbiota (hard fecal pellets) between allopatric subspecies of the European wild rabbit in wild populations and in captured individuals studied under captivity. We evaluated the influence of environmental and host-specific factors. The microbiome of wild rabbit populations reduced its heterogeneity under controlled conditions. None of the host-specific factors tested correlated with the microbiota composition. We only observed significant intra-group dispersion for the age factor. The most diverse microbiomes were rich in *Ruminococcaceae* potentially holding an enriched functional profile with dominance of cellulases and xylanases, and suggesting higher efficiency in the digestion of fiber-rich food. Conversely, low diversity gut microbiomes showed dominance of *Enterobacteriaceae* potentially rich in amylases. We preliminary noticed geographical variations in field populations with higher dominance of *Ruminococcaceae* in south-western than in north-eastern Spain. Spatial differences appeared not to be subspecies driven, since they were lost in captivity, but environmentally driven, although differences in social structure and behavior may also play a role that deserve further investigations. A marginally significant relationship between the *Ruminococcaceae*/*Enterobacteriaceae* ratio and potential life expectancy was observed in captive rabbits. We hypothesize that the gut microbiome may determine the efficiency of feeding resource exploitation, and can also be a potential proxy for life expectancy, with potential applications for the management of declining wild herbivorous populations. Such hypotheses remain to be explored in the future.

## Introduction

The physical condition of wild animals and their immunological performance determine the health status of wildlife populations (e.g^1^). Recently, it has been shown that physiological and immunological responses in animals are closely related to their gut microbiome^2^.

The structure and composition of the gut microbiome have co-evolved with their hosts^3,4^, particularly for dietary strategies and gut microbiota convergent adaptation^5^, but it can also be affected by others factors such as age, lifestyle or host health status, among others^6,7^. Substantial changes in the core gut microbiome may indicate intestinal disease or diminished host fitness^8,9^.

The consequences of host gut microbiome in wildlife physical condition have received little attention in the scientific literature to date. Most investigations have focused on the composition and changes of wildlife gut microbiota depending on environmental/external (e.g. habitat and diet) and host factors (see^10^ and references therein). The study of the relationship between gut microbiome structure and health status is particularly relevant in wildlife species that experience high mortalities, such as the European wild rabbit (*Oryctolagus cuniculus*). *O. cuniculus* is a keystone species that has important consequences for biodiversity management and for the fate of economic activity in rural areas where it is one of the main game species. Rabbit field mortality is mostly caused by predation and by the impact of viral diseases like myxomatosis and rabbit hemorrhagic disease (RHD). Although rabbit mortality has been reported to be as high as 85%^11^, there are strong variations in mortalities between years and populations (e.g.^12^). Because of the tight connections reported between physiological and immunological responses in animals and the gut microbiome^2^, we hypothesize that rabbit survival may be associated with gut microbiota composition, as this could lead to higher efficiencies in food assimilation and thus to a better physical condition. This might have important implications for rabbit management and conservation in the Iberian Peninsula, the species’ native range, and to other herbivorous animals. In recent decades, viral diseases have reduced rabbit populations in most natural areas to such a point that the species has been classified as endangered by the International Union for the Conservation of Nature^13^. However, some populations in farmland areas have exhibited substantial growth in recent years, causing severe damage to crops. In this context, developing new tools or indexes related to gut microbiota that may permit inferring the health status of wild rabbits could assist or facilitate decision-making processes for in situ population management.

**Figure 1:**
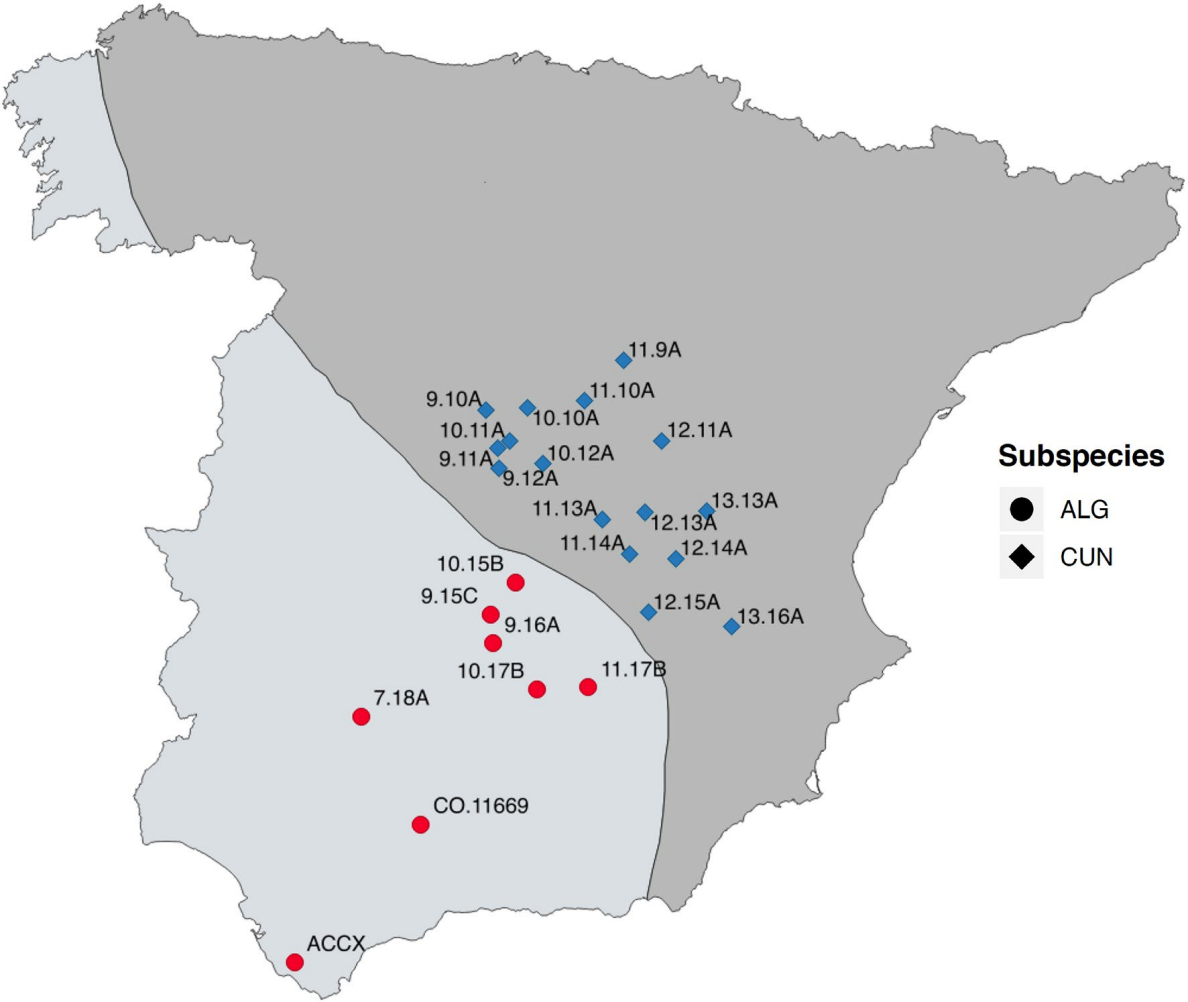
Map location and code for the field samples analyzed. The black diagonal line indicates the approximate limits for the geographical distribution of the subspecies *O. c. algirus* (southwest) and *O. c. cuniculus* (northeast). Hybrid individuals are also possible across the transition area of both populations. ACCX summarizes a set of 19 different individuals from one single *O. c. algirus* population. Map created with QGIS v. 3.10.10 (https://qgis.org/).

Genetic analyses have shown two subspecies for the European wild rabbit (*O. c. cuniculus* and *O. c. algirus)*, which evolved independently during the Quaternary glaciations in the Iberian Peninsula, approximately two million years ago^14^. Currently, the subspecies *O. c. algirus* is mainly restricted to the western portion of the Iberian Peninsula, whereas the subspecies *O. c. cuniculus* is distributed towards the northeast (Fig. 1). Virtually all rabbits that exist outside the Iberian Peninsula have been introduced by humans and belong to the subspecies *O. c. cuniculus*. Likewise, all domestic forms of the rabbit come from this same subspecies^14^. The hybridization between both subspecies rarely takes place not only because their distributions practically do not overlap (Fig. 1), but also due to their important genetic differences. In fact, the lower fertility of hybrids suggests an advanced stage of speciation^15^. Many other noticeable differences have been reported between rabbit subspecies in terms of morphology, parasitology, behavior or reproduction, amongst others^15,16^, and differences in social structure seem to exist between the two-rabbit subspecies, with *O. c. cuniculus* being potentially more sociable than *O. c. algirus* (R. Villafuerte, unpublished data). This has led some authors to even raise the possibility of considering *O. c. algirus* and *O. c. cuniculus* as already well-separated species^16^. This offers an excellent model to unravel the host-specific factors shaping the gut microbiota in nature.

We aimed to assess potential links between rabbit gut microbiota composition present in hard fecal pellets and the fate (survival) of individuals and to identify a biomarker index from field feces as a proxy of the gut microbiota functional performance. Additionally, we explored the microbiota of wild rabbits from geographically distant populations. Wild rabbits were studied under both controlled (i.e. captured wild individuals) and natural conditions (i.e. wild populations), including environmental (e.g. habitat, climate, etc.) and host-specific factors (e.g. subspecies, sex and age).

## Methods

### Sampling of wild rabbits

We studied the gut microbiota in pellets (hard fecal pellets) collected from both free-living populations and wild rabbits kept in captivity under controlled conditions. To preliminary explore geographic variations, 42 fecal samples from 24 wild rabbit populations in central-southern Spain (Andalusia, Castilla la Mancha, and Madrid, Fig. 1) were analyzed, covering the natural distribution areas of the two rabbit subspecies^17^. Wild individuals usually produce 10–20 fecal pellets per deposition in isolated piles in random locations^18^. Rabbits also accumulate pellets in special sites known as ‘latrines’, which are used to demarcate their social territory^19^. Field samples (3–4 hard fecal pellets) were selected mostly from small, isolated piles after recent defecation of a single individual when pellets were still slightly moist. In the case of site ACCX, samples were collected immediately after defecation from 19 captured individuals. The field surveys were conducted in August 2016.

We sampled wild rabbits kept in captivity to evaluate the effect of captivity on rabbit microbiota composition (Table S1). In addition, captive rabbits were used to test for potential effects on the composition of the microbiome of host-specific factors such as genetics, sex, age, and survival, once other variables were fixed (e.g. diet) which was not possible in field conditions. Captive rabbits were kept at the Wild Lagomorphs Research Centre (REGA-ES1402100962) of Córdoba, Spain. This center is officially registered for the breeding and use of experimental animals and is accredited by the regional government (Junta de Andalucía) in accordance with European Union guidelines on animal welfare. The experimental procedures were reviewed and approved by the Ethics Committee for Animal Research of the Spanish National Research Council (CSIC, Register Project CGL2013-43197-R) in accordance with the guidelines and regulations concerning animal welfare and experimentation set out under Spanish legislation. The facility consists of two different captive systems. In the first system, four semi-captive populations (two of each rabbit subspecies) were enclosed in separate fenced plots of 2500 m^2^. Each plot has 10 artificial warrens surrounded by a wire net (approx. 1 m high) connected to two rabbit traps. This system allows capturing a large proportion of the rabbit population inside the warren (i.e. 50–60%^20^). Rabbits were live-trapped in all warrens every two months and marked at their first capture with individually numbered ear tags and measured (sex, weight, tarsus and ear length). Since most rabbits weighed < 500 g at their first capture, it was possible to estimate their birth date using the linear formula that relates body weight and age for the species (e.g.^21^). In the other system, there were 42 smaller enclosures (2 × 3 m) with a wooden box to provide the rabbits refuge. Both the plots and the smaller enclosures had feeders and water suppliers for unrestricted access to food and water. The diet provided for all captive animals was exclusively based on pelleted food (CUNILAP, NANTA S.A., Spain) and natural plants were not allowed to grow in any of the enclosures. For the microbiome analyses, we captured 39 rabbits from the plots and 17 from the smaller enclosures. The rabbits were kept individually in 0.65 × 0.35 m metal cages under standard housing conditions for 24 h to collect a feces sample from each individual. After pellet collection, we released the rabbits (n = 39) into their original plots. Survival was monitored by means of the above-mentioned bimonthly capture protocol and periodic revisions (every 2–3 days) of rabbit carcasses in each plot. Animals that were captured at least once during 3 consecutive capture sessions were considered “live rabbits” and those whose carcasses were found during the periodic revisions were considered “dead rabbits”. In addition, we assigned the category “unknown fate” to animals that were never captured and whose remains were not found during the visual inspections of the plots. Most probably, these animals died underground inside the warren. We cannot rule out that sporadically some individuals may have been predated by raptors and carried over the fence.

### DNA analyses, sequencing and sequence processing

All rabbit feces were immediately kept in lysis buffer (40 mM EDTA; 50 mM Tris pH 8.3; 0.75 M sucrose) after collection and further frozen (− 20 °C) until DNA extraction^22^. Samples were thawed and submitted to smooth shaking for 30 min. Next, 200 µl of supernatant were used for the DNA extraction with the QIAamp DNA Stool kit (QIAGEN, Hilden, Germany). The variable V4 region of the bacterial and archaeal 16S rRNA gene was amplified with the F515 (5′-GTGCCAGCMGCCGCGGTAA-3′) and R806 (5′-GGACTACHVGGGTWTCTAAT-3′) primers using a paired-end 2 × 250 bp run^23^. The Illumina MiSeq platform was used for high-speed multiplexed SSU rRNA gene sequencing. PCR and sequencing were carried out according to the genomic core facilities and methods of the RTSF-MSU (Michigan State University, USA) (www.rtsf.natsci.msu.edu). The original dataset is available at the NCBI Sequence Read Archive under accession number SRP129755, with the metadata provided for all samples.

Raw sequences were processed using the UPARSE pipeline^24^. Samples with < 5000 reads were initially excluded. Prior to quality filtering, the dataset consisted of 3,265,734 sequences, around 73% ≥ Q30. After the read pairs were merged, the dataset was filtered by setting a maximum of 0.25 expected errors and discarding reads with higher error probabilities. The dereplicated reads were sorted by size, singletons were excluded, and chimeras were discarded with UCHIME following de novo and reference-based (against a ‘Gold’ reference database available for Chimera Slayer) strategies. Sequences were clustered to operational taxonomic units (OTUs) at a 97% identity threshold cut-off. Taxonomic assignment was carried out using the SILVA_128 reference database^25^ with the dereplicated version at a 99% sequence identity (SSURef_NR99_128_SILVA), and the identified chloroplasts and mitochondria were excluded. We usually run laboratory blank controls for DNA extraction, amplification, and sequencing. Blank are always less than two orders of magnitude of sequence-reads per sample than the rest of the field datasets and show highly differentiated taxonomic profiles. A literature survey of the main taxa found ruled out the presence of any substantial microbial contamination. The complete curated microbiome dataset included a total of 2142 OTUs and 99 samples.

### Statistical analyses

In order to minimize the potential effects of sequencing effort on the abundance-based analyses, the original OTU table was normalized by the cumulative-sum-scaling method (CSS^26^). We preliminary tested for the effect of sample sizes between categories before applying CSS normalization (Kruskal–Wallis test = 98, *P* = 0.481, on a variable considering different sample types, as feces from wild and captive individuals).

All statistical analyses were run in the R environment (R version 3.4.4, http://www.r-project.org/). Graphics were prepared with the *ggplot2* package^27^. Community ecology-related parameters were calculated using the *vegan* package^28^. To investigate monotonic association between numeric variables, Spearman’s rank-order correlation coefficient was used. Hypothesis contrast tests were carried out with the *stats* package and non-parametric tests were used when the assumptions for the parametric equivalents were not achieved. Analyses of similarities (ANOSIM) and the permutational multivariate analysis of variance using distance matrices (adonis test) were carried out based on 1000 permutations to test for differences between groups of samples belonging to different factor categories in the metadata. ANOSIM was only checked after discarding heteroscedasticity among those groups (permutest. betadisper function). For both analyses, we used Bray–Curtis distance matrices and took the value of the statistic to evaluate the test result since the probability (i.e. *P* value) was found to be highly dependent on the number of permutations in most of the cases. To calculate Faith’s phylogenetic diversity (PD) indices, the alignment was run with PyNAST using the file ‘core_set_aligned.fasta.imputed’ as template (available from http://greengenes.lbl.gov/). The tree was constructed with the ‘fasttree’ method. The predicted functional profile of microbial communities was inferred with the Tax4Fun package^29^ using default settings. We focused on KEGGs^30^ (Kyoto Encyclopedia of Genes and Genomes for enzymatic and metabolic pathways) related to enzymes involved in fiber and vegetable biopolymer^31^.

### Ethics approval and consent to participate

The experimental procedures were reviewed and approved by the Ethics Committee for Animal Research of the Spanish National Research Council (CSIC, Register Project CGL2013-43197-R) in accordance with the guidelines and regulations concerning animal welfare and experimentation set out under Spanish legislation.

## Results

**Figure 2:**
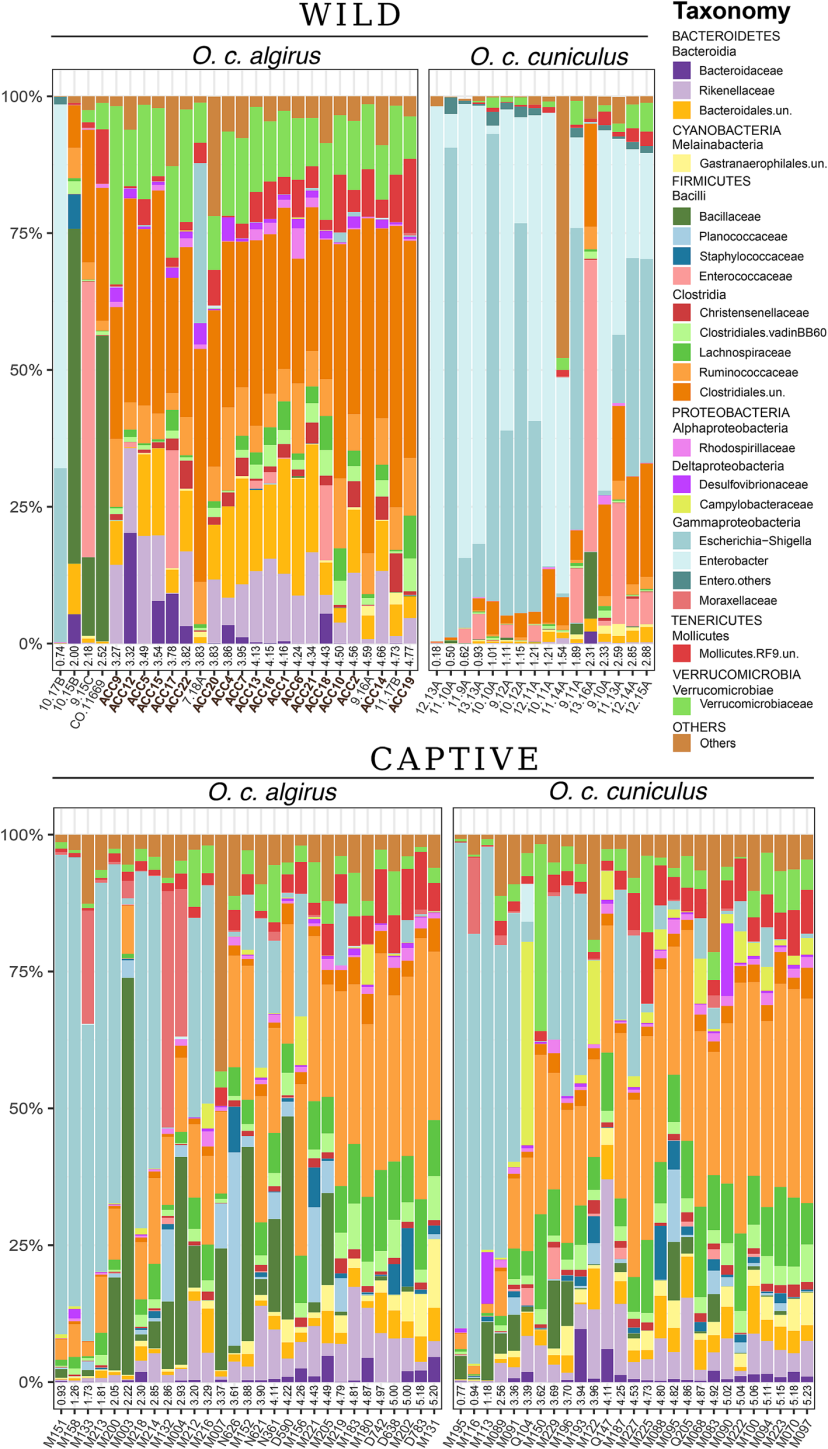
Bacterial taxonomic composition of the different gut microbiomes at the family level (only those with an average relative abundance > 0.5% are shown). Panels for wild (upper) and captive (lower) individuals sorted in ascending order according to the Shannon index. Each bar corresponds to one individual.

We noticed high variations in the gut microbiota of the rabbits studied, with some individuals dominated (from ~ 50% to almost 100% of relative abundance) by *Enterobacteriaceae* and others with a high dominance (~ 30%) of *Ruminococcaceae* (Fig. 2). *Enterobacteriaceae* were almost exclusively *Escherichia–Shigella* in the captive animals, while the genus *Enterobacter* was the most frequent in the field. Additionally, *Bacillaceae*, *Enterococcaceae*, *Planococcaceae* and *Moraxellaceae* occasionally appeared with a high frequency in some individuals (Fig. 2). Considering all samples (together field and captive rabbits), we found a significant correlation between the ecological diversity of the microbiota (i.e. Shannon index) and the abundance of the most relevant bacterial taxa both direct (*Ruminococcaceae,* Spearman’s rank correlation, rho = 0.85, *P* value < 0.01) and indirect (*Enterobacteriaceae,* Spearman’s rank correlation, rho = − 0.61, *P* value < 0.01) (Fig. S1). *Ruminococcaceae* and *Lachnospiraceae* were the taxa with the highest Spearman correlation coefficient. Indeed, *Ruminococcaceae* were among the dominant taxa in the most diverse microbiotas. Conversely, *Enterobacteriaceae* was the only taxon that showed a negative slope between relative abundance and microbiota diversity.

**Figure 3:**
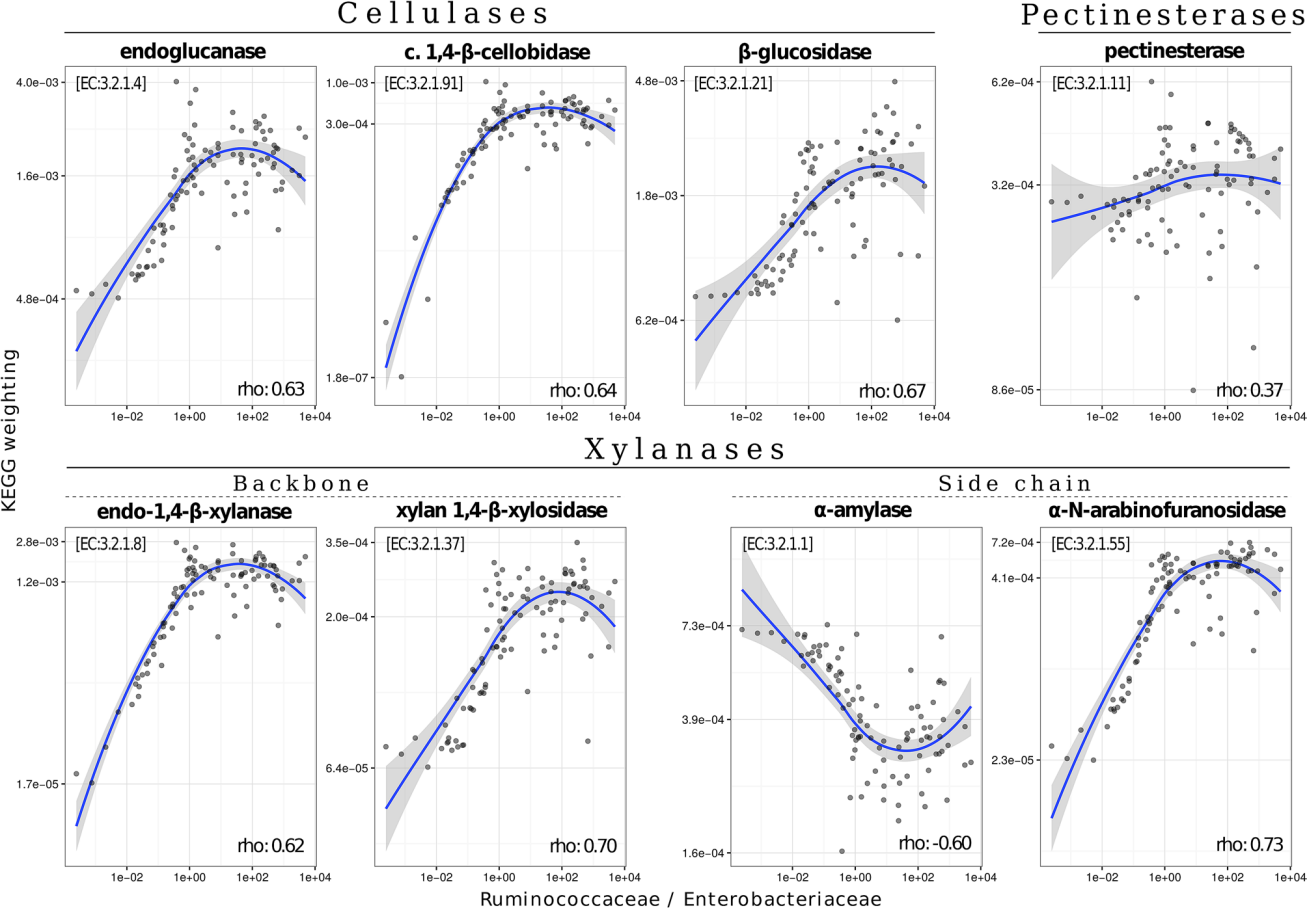
Correlation between the weighted importance of inferred enzyme-specific genes (i.e. KEGGs) (y-axis) related to vegetable fiber degradation and the *Ruminococcaceae*/*Enterobacteriaceae* ratio (x-axis). KEGG weighting values refer to predicted adjusted abundances in the entire gut microbial community. Spearman’s rank-order coefficients were significant in all cases (*P* < 0.001).

**Figure 4:**
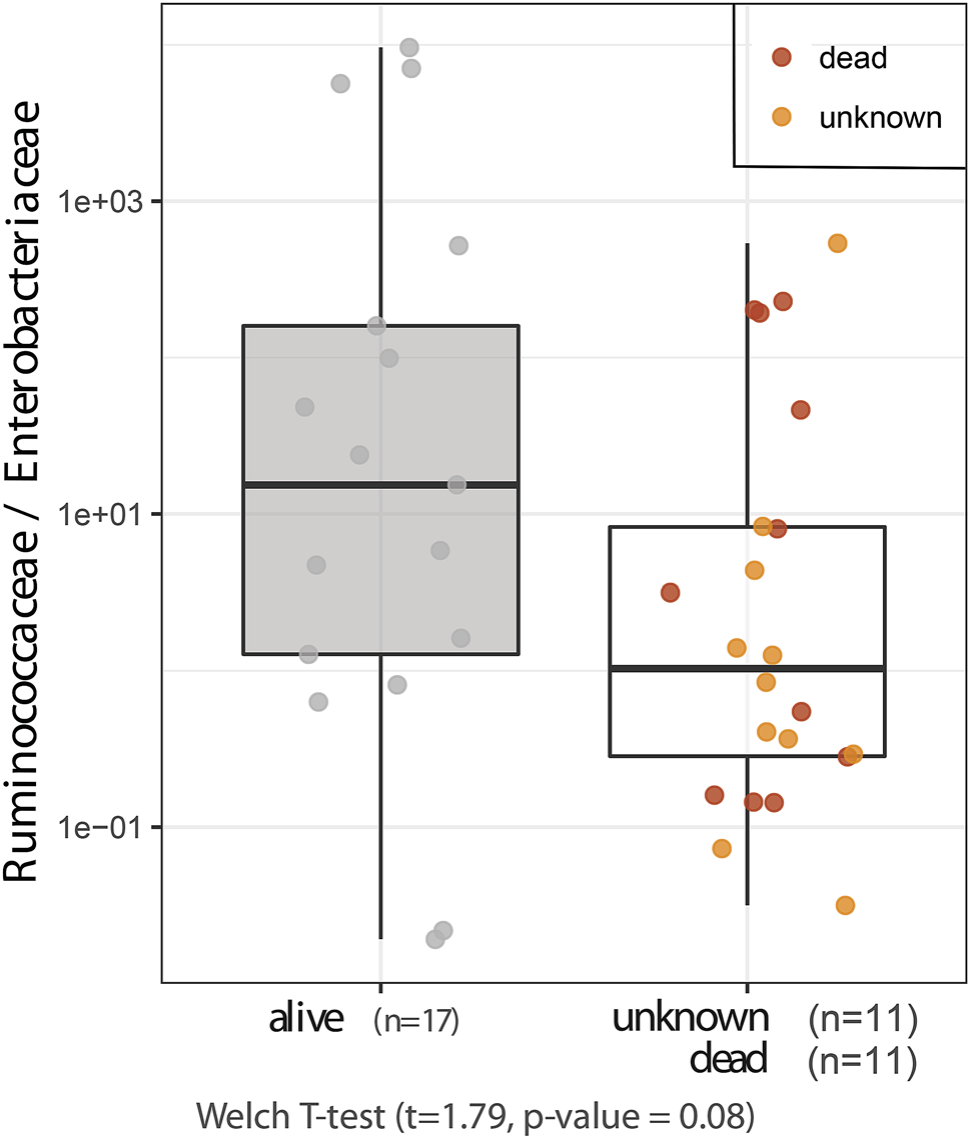
Comparison of the *Ruminococcaceae*/*Enterobacteriaceae* ratio according to life expectancy for captive animals (log transf. data). Unknown fate refers to animals that were never captured and whose carcasses were not found during the visual inspections of the plots. Most probably, these animals died underground inside the warren (see main text).

A strong and significant positive correlation was found between the weighted proportion of predicted KEGGs related to the enzymatic degradation of raw vegetable (fibrous) matter and the dominance of *Ruminococcaceae* vs. *Enterobacteriaceae* (Fig. 3). Conversely, the predicted α-amylase dominance in the gut was closely associated with a higher abundance of *Enterobacteriaceae*. We observed a marginally significant positive relationship (n = 39, Welch t-test, *P* = 0.08) between life expectancy and the *Ruminococcaceae*/*Enterobacteriaceae* index in semi-captive rabbits (Fig. 4). We included in this analysis individuals with unknown fate that most probably died underground inside the warren. When these animals were removed from the analyses, it remained still marginally significant (n = 28, *P* = 0.09).

**Figure 5:**
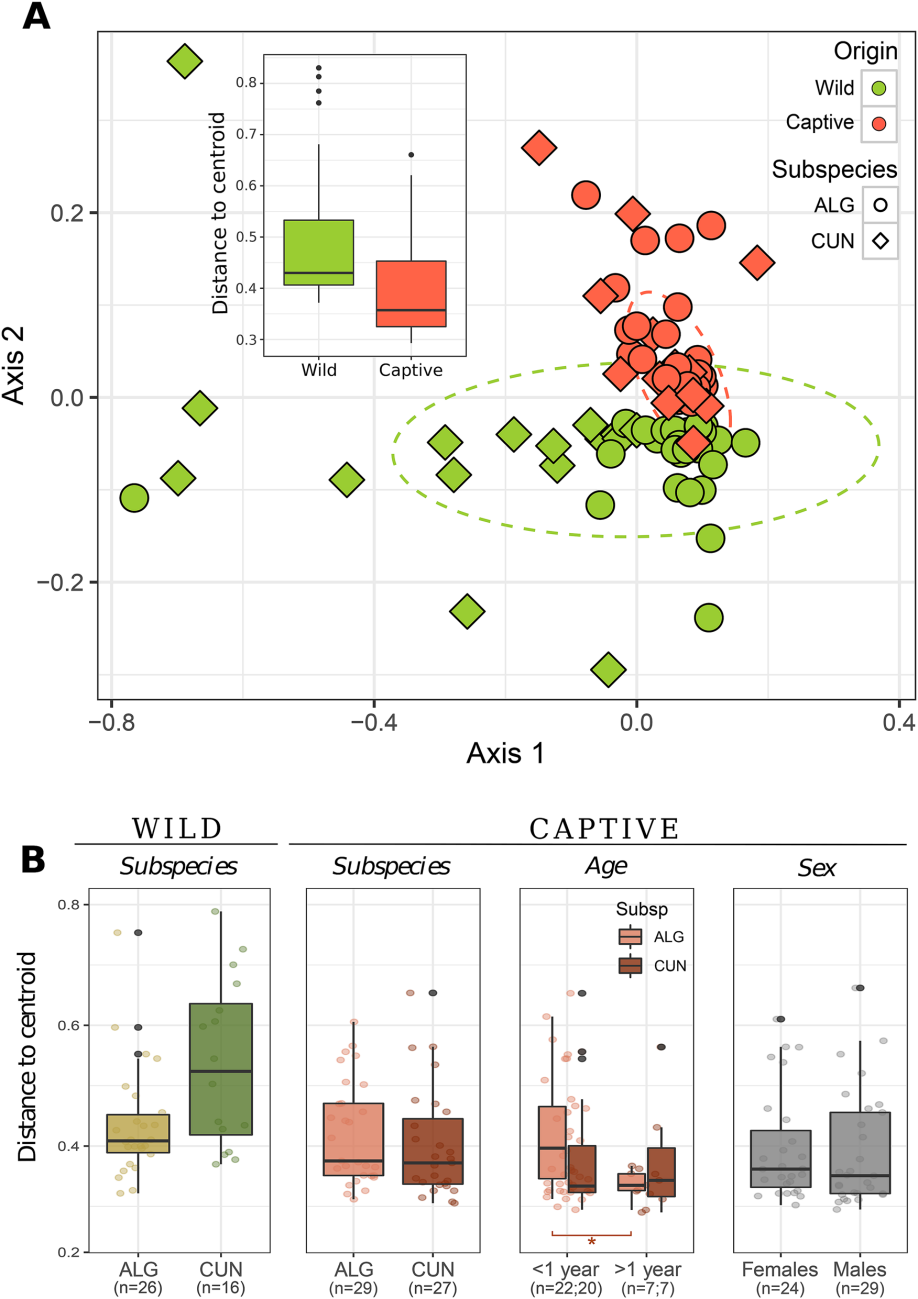
(**A**) Non-metric multidimensional scaling ordination analysis (NMDS) showing the dissimilarity between gut microbiomes according to origin (Wild, Captive) for *O. c. algirus* (ALG), and *O. c. cuniculus* (CUN). Confidence ellipses at the 95% confidence level. The boxplot in the figure shows the heterogeneity according to the distance to centroid. Wild samples showed higher intra-group dispersion (Wilcoxon test, W = 1847, *P* < 0.01). (**B**) Centroid distances between samples of each category for the three factors under study (subspecies, ageandsex). Statistical analyses run in R (R version 3.4.4, http://www.r-project.org/).

Microbiota gut composition showed a high variability between wild individuals from different locations, but this heterogeneity was lost in captivity. In fact, gut communities showed significant differences between field and captive populations (adonis, R^2^ = 0.09, *P* < 0.01), with a smaller intra-group dispersion in the microbiota of captive individuals (Betadisper, F = 6.54, df = 1, *P* < 0.05) (Fig. 5, inlet in panel A). Variations in gut microbiota heterogeneity were observed between field populations in the distribution areas of both rabbit subspecies (adonis, R^2^ = 0.12, *P* < 0.01), with individuals surveyed in the *O. c. cuniculus* distribution area showing more dissimilar gut communities than those in the *O. c. algirus* distribution area (Betadisper: F = 20.46, df = 1, *P* < 0.01) (Fig. 5, panel B-left). In addition, gut communities from field populations in the *O. c. algirus* distribution area were slightly, but significantly, different than those found in the *O. c. cuniculus* distribution area (adonis: R^2^ = 0.03, *P* < 0.01). The same trend was observed when alpha diversity parameters were analyzed (Richness, Shannon and PD, Fig. S2). Overall, the alpha diversity of wild populations in the *O*. *c*. *cuniculus* distribution area was lower than that of animals in the *O. c. algirus* distribution area and of those kept in captivity (Fig. S2). Again, differences in microbial gut heterogeneity and alpha diversity between subspecies were lost when wild rabbits were kept in captivity (Fig. 5, panel B, Fig. S2).

**Figure 6:**
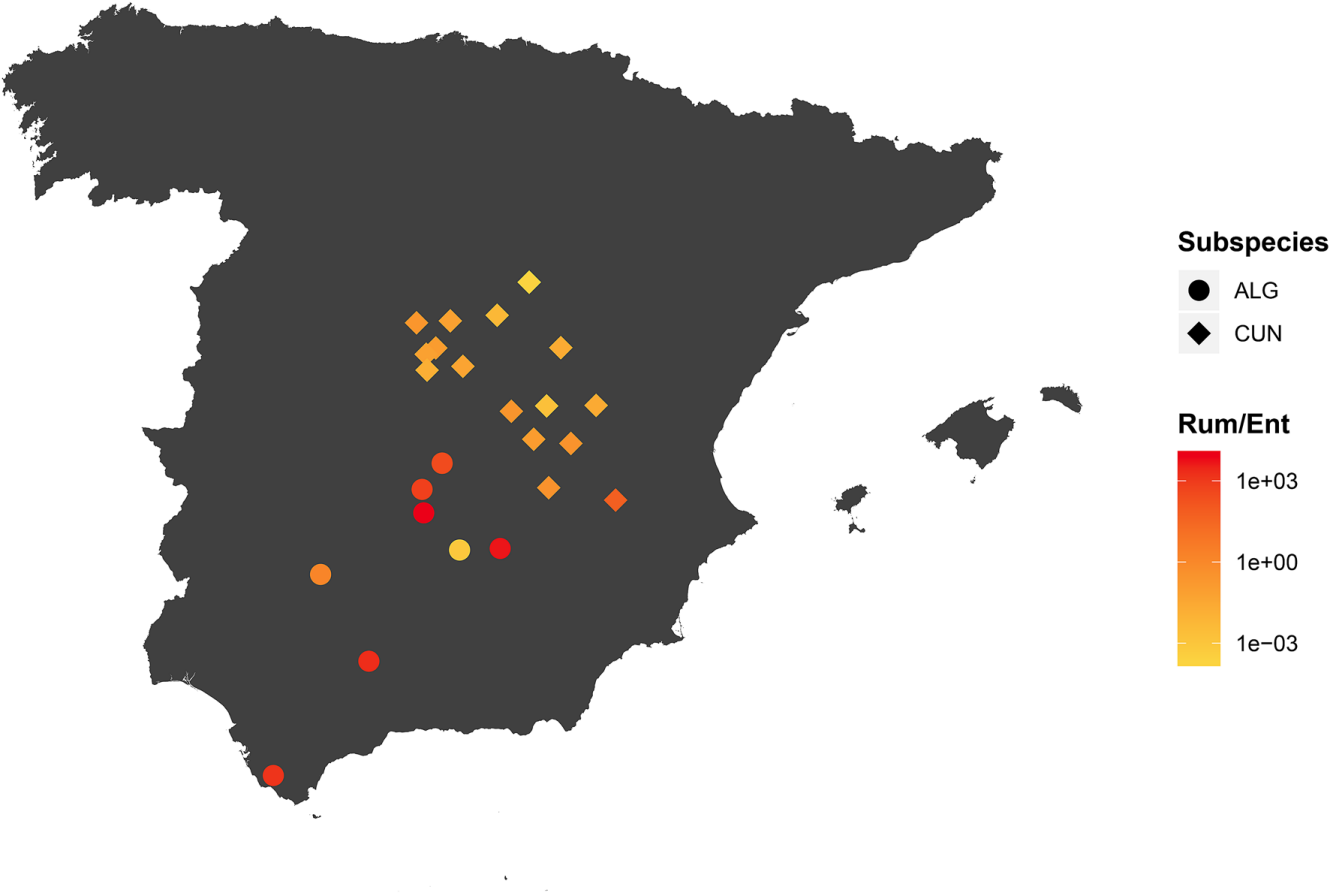
Map of the Iberian Peninsula with the location of field samples and the *Ruminococcaceae*/*Enterobacteriaceae* ratio. For the ACCX *O. c. algirus* population (see Fig. 1), the mean value for 19 independent individuals is shown. Map created with R packages rgdal, v.1.4-8 (https://cran.r-project.org/web/packages/rgdal/), and maptools, v. 0.9-9 (https://cran.r-project.org/web/packages/maptools/maptools.pdf). Spain shapefile map source: GADM, v3.6 (https://gadm.org/download_country_v3.html).

**Figure 7:**
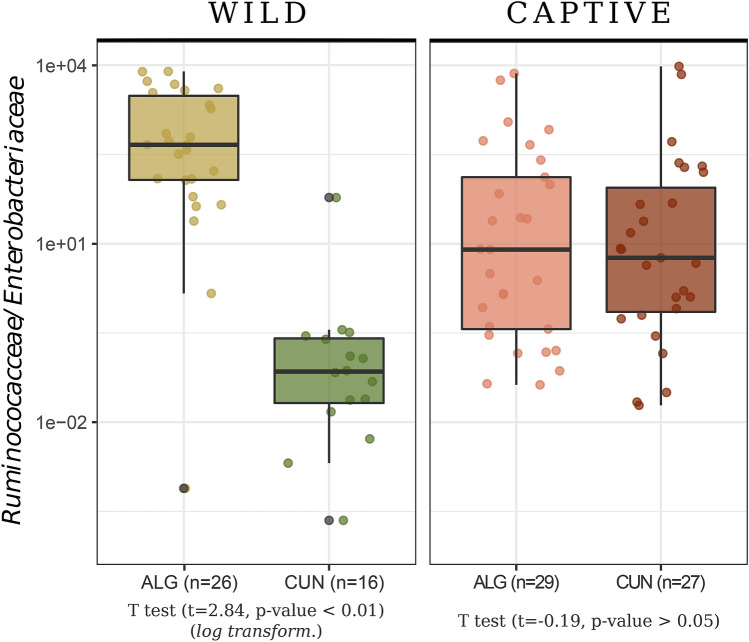
Comparison of the gut functional enrichment index (ratio between relative abundance of *Ruminococcaceae* and *Enterobacteriaceae*) between subspecies for captive and wild groups. ALG: *O. c. algirus.* CUN: *O. c. cuniculus*.

Furthermore, the microbiota of rabbits from field populations in the distribution area of *O*. *c*. *algirus* was, in general, more diverse (Fig. S2) and showed a dominance of *Ruminococcaceae* (0.311 ± 0.061), *Verrucomicrobiaceae* (0.139 ± 0.056), *Rikenellaceae* (0.118 ± 0.065) and unclassified *Bacteroidales* (0.108 ± 0.05) (Fig. 2, upper panel). Conversely, *Enterobacteriaceae* dominated the gut microbiota of field populations in the *O. c. cuniculus* distribution area in most cases. Interestingly, we also observed spatial differences in the *Ruminococcaceae*/*Enterobacteriaceae* index with southwestern individuals (i.e. in the *O. c. algirus* distribution area) showing the taxonomically and functionally more diverse microbiomes (Fig. 6), which, according to this gut indicator, may potentially contain the healthiest populations. In fact, we found a predicted higher functional versatility for fiber digestion in field populations in the *O. c.algirus* distribution area (Wilcoxon test, W = 395, *P* < 0.01)*,* but differences between subspecies were again lost under captivity (Fig. 7).

Finally, when we analyzed under controlled conditions whether host-specific variables (i.e. rabbit subspecies, age and sex) correlated with the microbiota composition, no significant differences were found between sexes or age groups (sex Betadisper, F = 0.05, df = 1, *P* = 0.81; age Betadisperser, F = 2.67, df = 1, *P* = 0.11; Fig. 5, panel B). We only observed differences in dispersion for the age factor between subspecies (Betadisper, age: F = 8.01, *P* < 0.001; Fig. 5, panel B). Thus, higher dispersion was observed in young individuals (< 1 year) of *O. c. algirus* than in *O. c. cuniculus* (*t*-test, t statistic = 6.69, *P* < 0.01). In contrast, we did not observe differences for the sex factor between subspecies (*t*-test, t statistic = 0.15, *P* = 0.7).

## Discussion

In this study we carried out a detailed description of the variation in gut microbiota observed between allopatric subspecies of the European wild rabbit, a keystone species in Iberian Mediterranean areas. Our study explored multiple parameters potentially affecting the host—microbial composition, inferred functional indicators, and survival for wild rabbits captured and studied under captivity. We preliminarily identified a gut bacterial index for the European wild rabbit to connect life expectancy and gut functional performance with potential applications in management and conservation practices of wild populations that deserves further investigations, mainly to substantiate the field observations. The study demonstrates clear differences in microbiome composition in the distribution areas of the two rabbit subspecies in the field. We detected spatial patterns although the differences appeared not to be subspecies driven (since they were lost in captivity) but environmentally driven.

Like many other lagomorph species, European rabbits have evolved several mechanisms that enable them to maximize the assimilation of nutrients; in fact, such mechanisms allow rabbits to cope with forage with high-fiber content (i.e.^32^). Rabbits are monogastric herbivores with a colonic separation that allows them to quickly excrete poorly digestible large particles whilst retaining fine food particles in the caecum for a sufficiently long period to allow for fermentation and microbial reproduction^33^. As a result, two feces types are produced: ‘hard’ ones, which are composed of poorly digestive particles, and ‘soft’ ones, which correspond to the material fermented in the caecum. Rabbits take full advantage of both the final fermentation products and many of the microbial proteins present in these soft feces through their re-ingestion immediately after excretion from the anus (i.e. cecotrophy^33^). Interestingly, it has been reported that microbiota differs between hard and soft feces^34^. Additionally, it may be presumed that some specific gut bacterial taxa are involved in rabbits’ ability to digest different types of food. In the present work, we focused on the microbial populations present in hard faces as a part of the total gut microbiota and as a valuable source of information but we did not characterize the total gut microbiome of wild rabbits.

The presence of certain bacterial taxa in the gut microbiota has been reported as healthful for the host, while other groups are known to be unfavorable. For example, members of the family *Ruminococcaceae*, such as *Faecalibacterium prausnitzii*, have been shown to be beneficial for intestinal health, whereas *Enterobacteriaceae*, like *Escherichia coli,* are potentially pathogenic gut commensals for the mammalian host^35,36^. *Ruminococcaceae* are often abundant in the gut microbiota of different herbivores (see Table S2 and references therein). In the present study, we demonstrated that there are important differences in the gut microbiota composition of different wild rabbit populations, with a spatially heterogeneous in situ distribution of the *Ruminococcaceae*/*Enterobacteriaceae* index that may unveil important functional variability among different individuals.

*Ruminococcaceae* and *Lachnospiraceae* are bacterial taxa specialized in decomposing complex plant materials (i.e. cellulosic and recalcitrant matter from vegetables)^31^. They can also perform acetogenic metabolism in the gut^37^ and some of these bacteria can also produce butyrate^38^, a biomolecule linked to healthy gut functioning^39^. In addition, the genus *Ruminoccocus* is also known for its efficient role in large polysaccharide (cellulosic) degradation^40^. Conversely, *Escherichia–Shigella* and *Enterobacter* are recognized in mammals as being both potentially pathogenic gut commensals^35,36,41^ and temporal intestinal colonizers^42^ which also become abundant after host-mediated inflammation in response to the presence of pathogens^43^. The dominance of *Enterobacteriaceae* within the gut microbial community has been reported as being harmful, since they trigger an insufficient intestinal colonization resistance against other pathogens^44^. In addition, *Enterobacteriaceae* facilitate the propagation of pathogenicity-associated genes between pathogenic and commensal strains, thus exacerbating intra-family conjugation rates^36^. Finally, an association has also been observed between gut microbiota composition and liver disease in humans, with strong decreases in the *Ruminococcaceae*/*Enterobacteriaceae* ratio as compared to healthy individuals^45^. Altogether, there is a plenty of evidence to support that the *Ruminococcaceae*/*Enterobacteriaceae* index captures rabbit gut alteration (dysbiosis), similar to other indexes typically used in human health studies^46^. Indeed, we observed partially significant differences in rabbit life expectancy related to the *Ruminococcaceae*/*Enterobacteriaceae* index although this finding deserves further investigations to be fully substantiated. Rabbit distribution in the field is very patchy and, paradoxically, high density populations commonly occur alongside others which are depleted^16^. We hypothesize that gut microbiota composition may be connected to these distributions as occurs in other lagomorph species with a similar functional role, such as the pika (*Ochotona curzoniae*) in the Tibetan Plateau ecosystem. Pika population density has been positively related to the alpha diversity of gut microbial communities and to the abundance of some microbes such as *Ruminococcaceae*, *Lachnospiraceae* and *Staphylococcaceae*^47^. Additional research is however needed to properly address whether or not the gut microbiota has a substantial role on rabbit’s life expectancy.

In the present study, we reported for the first-time geographical variations in wild rabbit gut microbiome. For example, *Enterobacteriaceae* tended to be overrepresented in northern rabbit populations. Similarly, the abundance of these potential pathogenic bacteria varies significantly between brown hare (*Lepus europaeus*) populations in central Europe^41^. It is very likely that geographical variations in rabbit microbiota are explained by environmental factors like habitat quality and availability of food resources^48^. Moreover, it is expected that the wild rabbit gut microbiome contains specific bacterial strains that are well adapted to the rabbit diet and capable of degrading and obtaining energy and resources from vegetable fibers and biopolymers. European rabbits are small, selective and opportunistic grazers that usually feed on low-fiber herbaceous plants and seek specific weeds and grasses of certain taxa. However, they are also able to exploit all kind of resources available in a high variety of feeding grounds (i.e. woody parts of herbs and scrubs, tree roots and seeds), which explains their ecological plasticity and capacity to subsist in many different habitats^49^. Accordingly, rabbits’ diet composition varies spatially depending on the availability of plant species, as well as their phenological development, palatability and nutritive value (e.g.^50,51^). Therefore, variations in rabbit gut microbiome could be expected between populations that occur in different environments and thus feed on different resources. In general, rabbits prefer to feed on low-fiber herbaceous plants when available, but they can easily adapt to any food source when needed, as shown by the habitat quality of both their native range and the new areas where the species has been successfully introduced^51^. We found a significant positive correlation between the potential presence of KEGGs coding for enzymes involved in this core digestive function and the *Ruminococcaceae*/*Enterobacteriaceae* index. *Enterobacteriaceae* are α-amylase producers^52^ and although this enzyme is related to the degradation of side chains of xylan^53^, it is mainly involved in starch digestion^54^ but less efficient in degrading more fibrous material. Therefore, individuals with enriched *Enterobacteriaceae* in the gut microbiota may have more difficulties to fully profit from the energy and resources present in more heterogeneous and fibrous diets, potentially compromising their ecological fitness. If this is the case, most rabbit populations sampled in northern areas would be potentially more vulnerable than those sampled in the south. Because of the limitations of our field study and the fact that we deal with correlational observations and not causation, this result should be confirmed with a larger and more complex field survey in the future.

Given that intestinal core microbiomes have co-evolved with their hosts^3,4^, microbiome composition relies not only on external factors like diet and environment, but also on internal factors, such as ranges in stomach pH or host genotype^55^. Our results showed substantial differences in the gut microbiota between wild rabbit populations located in the distribution areas of the two-existing subspecies, but differences between subspecies were diluted under captivity. Thus, captivity (and/or diet) tended to significantly reduce microbiota heterogeneity. This result agrees with very recent studies showing that providing natural diets in captivity facilitates both retention of native gut microbiomes of captive animals and the potential for a better adaptation of relocated individuals^56,57^. These studies generally convey that the captive environment modifies the composition of the original wild healthy animal microbiota to a variable extent.

It has been reported that group living and social interactions may also predict gut microbial composition^58^. Rabbits usually live in groups associated with their warrens^59^ but rabbit sociality often varies among populations depending on the proportion of the so-called ‘surface dwellers’^60^. In this sense, strong differences in social behavior seem to exist between the two-rabbit subspecies, with *O. c. cuniculus* being potentially more sociable than *O. c. algirus* (R. Villafuerte, unpublished data). In principle, individuals with more social partners should exhibit higher gut microbial diversity than socially isolated animals^61^ but this was not the case observed as a whole here. Although environmental factors may act as a major component in shaping most of the rabbit microbiome composition (see above), we cannot completely rule out that some of the differences in microbiota diversity found in the field may be related to idiosyncratic variations in sociality between rabbit subpopulations and genetics. Higher social interactions could be also involved in spreading and maintenance of harmful intestinal colonizers as *Enterobacteriaceae* at populational level. This might also explain how captivity reduced the heterogeneity among populations and the prevalence of *Enterobacteriaceae* in some individuals (also in *O. c. algirus*). Certainly, the connections microbiome-behavior in wild animals deserve further investigations.

Overall, we have reported for the first-time the gut microbiota in wild rabbit populations in situ combined with captive individuals studied under controlled conditions. Our results show that microbiota composition varies greatly between populations in the field. The differences did not seem to be subspecies driven but strongly shaped by the environment (probably diet or other unmeasured factors). These results could have potential implications for wild rabbit management in the Iberian Peninsula, where thousands of rabbits are released every year either for shooting or as additional prey for rabbit-dependent predators^62^. These continuous rabbit restocking operations have usually had little success, which has been traditionally attributed to the lack of adaptation to the new environment^63^. It is likely that the functional performance of rabbit microbiome is involved in this low restocking success^57^. From the results obtained here, we hypothesize that the gut microbiome may determine the efficiency of feeding resource exploitation, and can also be a potential proxy for life expectancy, with potential applications for the management of declining wild herbivorous populations. Further investigations may substantiate this very promising line of research.

## Supplementary information

Supplementary Information.

## Data Availability

The original dataset is available at the NCBI Sequence Read Archive SRP129755.
