# Supplementary material for: Individual fate and gut microbiome composition in the European wild rabbit (Oryctolagus cuniculus)
Source: Sci Rep. 2021 Jan 12;11:766. doi: 10.1038/s41598-020-80782-4 (PMC7804928; doi:10.1038/s41598-020-80782-4)

SUPPLEMENTARY INFORMATION
(2 Tables and 2 Figures)

Individual fate and gut microbiome composition in the European wild rabbit (*Oryctolagus cuniculus*)

Gerard Funosas, Xavier Triadó-Margarit, Francisca Castro, Rafael Villafuerte, Miguel Delibes-Mateos, Carlos Rouco, Emilio O. Casamayor*

E-mail: casamayor@ceab.csic.es

Table S1. Ancillary data for captive rabbits from different plots and cages in the Wild Lagomorph Investigation Center (code R.E.G.A. 021CO00962): *subspecies* ('CUN': *O. c. cuniculus*, 'ALG': *O. c. algirus*), *sex* ('F': female, 'M': male), *weight* (g), *tarsus* (mm), *BMI* (Body Mass Index: weight/tarsus), *age* (years), *fate* ('unk': unknown, see main text). Biometric variables (weight, tarsus, body mass index) and fate only available for rabbits in plots.

sample	Captive	subsp	sex	plot	warren	weight	tarsus	BMI	age	fate	sample	Captive	subsp	sex	cage	age
M068	PLOT	CUN	M	1	2	1164	58	20,07	<1	unk.	D590	CAGE	ALG	F	19	<1
M070	PLOT	CUN	M	1	5	1125	60	18,75	<1	alive	D638	CAGE	ALG	F	19	>3
M083	PLOT	CUN	M	1	NA	1218	60	20,30	2-3	alive	D742	CAGE	ALG	F	19	2-3
M088	PLOT	CUN	M	1	8	1055	62	17,02	<1	dead	D783	CAGE	ALG	F	19	2-3
M089	PLOT	CUN	M	6	4	1124	61	18,43	<1	dead	M003	CAGE	ALG	F	20	<1
M090	PLOT	CUN	M	6	2	1284	61	21,05	<1	alive	M004	CAGE	ALG	F	20	<1
M091	PLOT	CUN	M	6	7	1125	58	19,40	<1	dead	M007	CAGE	ALG	F	20	<1
M094	PLOT	CUN	F	6	1	1035	60	17,25	<1	alive	M131	CAGE	ALG	M	15	<1
M095	PLOT	CUN	F	6	1	1029	61	16,87	<1	alive	M132	CAGE	ALG	F	11	<1
M097	PLOT	CUN	F	6	9	1129	60	18,82	<1	dead	M133	CAGE	ALG	M	15	<1
M100	PLOT	CUN	M	6	6	1257	61	20,61	<1	dead	M151	CAGE	ALG	M	15	<1
M113	PLOT	CUN	F	6	5	1085	62	17,50	<1	alive	M152	CAGE	ALG	F	11	<1
M116	PLOT	CUN	M	6	9	1180	60	19,67	<1	alive	M200	CAGE	ALG	F	11	<1
M122	PLOT	CUN	F	6	2	1239	61	20,31	2-3	alive	M202	CAGE	ALG	M	12	<1
M150	PLOT	CUN	M	1	9	1171	59	19,85	2-3	dead	M205	CAGE	ALG	M	12	<1
M156	PLOT	ALG	F	3	5	975	58	16,81	<1	unk.	N361	CAGE	ALG	F	4	1-2
M158	PLOT	ALG	M	3	8	994	53	18,75	<1	unk.	N626	CAGE	ALG	F	2	1-2
M180	PLOT	ALG	M	8	6	1060	55	19,27	<1	alive						
M183	PLOT	ALG	F	8	7	1210	54	22,41	1-2	alive						
M187	PLOT	CUN	M	6	2	1194	60	19,90	<1	alive						
M193	PLOT	CUN	F	6	3	1055	62	17,02	<1	dead						
M195	PLOT	CUN	F	6	5	1266	60	21,10	<1	unk.						
M196	PLOT	CUN	F	6	5	1198	62	19,32	<1	alive						
M212	PLOT	ALG	M	3	10	977	50	19,54	<1	unk.						
M213	PLOT	ALG	M	3	10	962	54	17,81	<1	dead						
M214	PLOT	ALG	M	3	8	961	55	17,47	<1	unk.						
M216	PLOT	ALG	M	3	6	998	55	18,15	<1	unk.						
M218	PLOT	ALG	M	3	4	933	52	17,94	<1	dead						
M219	PLOT	ALG	M	3	3	1085	55	19,73	<1	dead						
M221	PLOT	ALG	M	8	3	950	54	17,59	<1	unk.						
M222	PLOT	CUN	M	1	2	1040	60	17,33	<1	unk.						
M223	PLOT	CUN	F	1	10	1080	57	18,95	1-2	dead						
M225	PLOT	CUN	F	1	7	1000	57	17,54	<1	alive						
M227	PLOT	CUN	M	1	8	1457	58	25,12	<1	unk.						
M229	PLOT	CUN	M	6	2	1140	61	18,69	<1	alive						
N621	PLOT	ALG	M	8	7	958	53	18,08	1-2	unk.						
Q104	PLOT	CUN	F	6	8	1374	61	22,52	1-2	alive						
Q147	PLOT	CUN	F	6	6	1199	59	20,32	1-2	alive						
Q205	PLOT	CUN	F	1	8	1203	58	20,74	1-2	alive						

Table S2. Published data for the relative abundance of *Ruminococcaceae* vs. *Enterobacteriaceae* in different animal gut microbiomes.

Diet	Animal	Status	Samples origin (number)	% *Ruminococcaceae* vs. % *Enterobacteriaceae*	Reference
Herbivore	New Zealand white rabbit (*O. cuniculus*)	Captive, healhty	Faeces (20)	13.8% vs. ~0%	Eshar & Weese, 2014
	Rex rabbit (*O. cuniculus*)	Captive	Faeces (20)	~15% vs. ~2%	Zeng et al., 2015
	Cattle (*Bos* spp.)	Captive, healthy	Faeces (30)	~35% vs. <1%	Shanks et al., 2011
	White rhinoceros (*Ceratotherium simum*)	Semi-captive, healthy	Faeces (4)	19.5% vs. ~0%	Bian et al., 2013
	Horse (*Equus ferus caballus)*	Captive, healthy	Faeces (6)	68.1% Firmicutes vs. 10% Proteobacteria	Costa et al., 2012
		Captive, unhealthy	Faeces (4)	30% Firmicutes vs. 18% Proteobacteria	
	Chicken (*Gallus gallus domesticus)*	Captive	Caecum (40)	~13% vs. <3%	Danzeisen et al., 2011
	Gopher tortoise (*Gopherus polyphemus*)	Wild	Faeces (46)	~ 38% Firmicutes vs. <3%	Yuan et al., 2015
Insectivore	Waterhouse's leaf-nosed bat *(Macrotus waterhousii)*	Wild	Intestine (22)	0.56% vs. 20.8%	Phillips et al., 2012
Hematophagous	Common vampire bat (*Desmodus rotundus*)	Wild	Intestine (3)	0.49% vs. 4.4%	

References

Bian, G., Ma, L., Su, Y., & Zhu, W. (2013). The microbial community in the feces of the white rhinoceros (*Ceratotherium simum*) as determined by barcoded pyrosequencing analysis. *PLoS One,* 8(7): e70103. doi: 10.1371/journal.pone.0070103

Costa, M. C., Arroyo, L. G., Allen-Vercoe, E., Stämpfli, H. R., Kim, P. T., Sturgeon, A., & Weese, J. S. (2012). Comparison of the fecal microbiota of healthy horses and horses with colitis by high throughput sequencing of the V3-V5 region of the 16S rRNA gene. *PLoS One,* 7(7): e41484. doi: 10.1371/journal.pone.0041484

Danzeisen, J.L., Kim, H.B., Isaacson, R.E., Tu, Z.J., & Johnson, T.J. (2011). Modulations of the chicken cecal microbiome and metagenome in response to anticoccidial and growth promoter treatment. *PLoS One,* 6(11): e27949. doi: 10.1371/journal.pone.0027949

Eshar, D. & Weese, J. S. (2014). Molecular analysis of the microbiota in hard feces from healthy rabbits (*Oryctolagus cuniculus*) medicated with long term oral meloxicam. *BMC Veterinary Research,* 10, 62. doi: 10.1186/1746-6148-10-62

Phillips, C. D., Phelan, G., Dowd, S. E., McDonough, M. M., Ferguson, A. W., Delton, H. J., ... & Baker, R. J. (2012). Microbiome analysis among bats describes influences of host phylogeny, life history, physiology and geography. *Molecular Ecol*ogy 21, 2617–2627. doi: 10.1111/j.1365-294X.2012.05568.x

Shanks, O.C., Kelty, C. A., Archibeque, S., Jenkins, M., Newton, R.J., McLellan, S. L., Huse, & S. M., Sogin, M. L. (2011). Community structures of fecal bacteria in cattle from different animal feeding operations. *Applied Environmental Microbiology,* 77, 2992-3001. doi: 10.1128/AEM.02988-10

Yuan, M. L., Dean, S. H., Longo, A. V., Rothermel, B. B., Tuberville, T. D. & Zamudio, K. R. (2015). Kinship, inbreeding and fine-scale spatial structure influence gut microbiota in a hindgut-fermenting tortoise. *Molecular Ecology,* 24: 2521-2536. doi:10.1111/mec.13169

Zeng, B., Han, S., Wang, P., Wen, B., Jian, W., Guo, ... & Li, Y. (2015). The bacterial communities associated with fecal types and body weight of rex rabbits. *Scientific Reports* 5(1), 9342. doi: 10.1038/srep09342

Fig. S1. Correlation between the relative abundance of *Ruminococcaeae* and *Enterobacteriaceae* and community Shannon index, respectively, for each sample. Spearman's Rank order coefficients strongly significant (*P* <0.001).

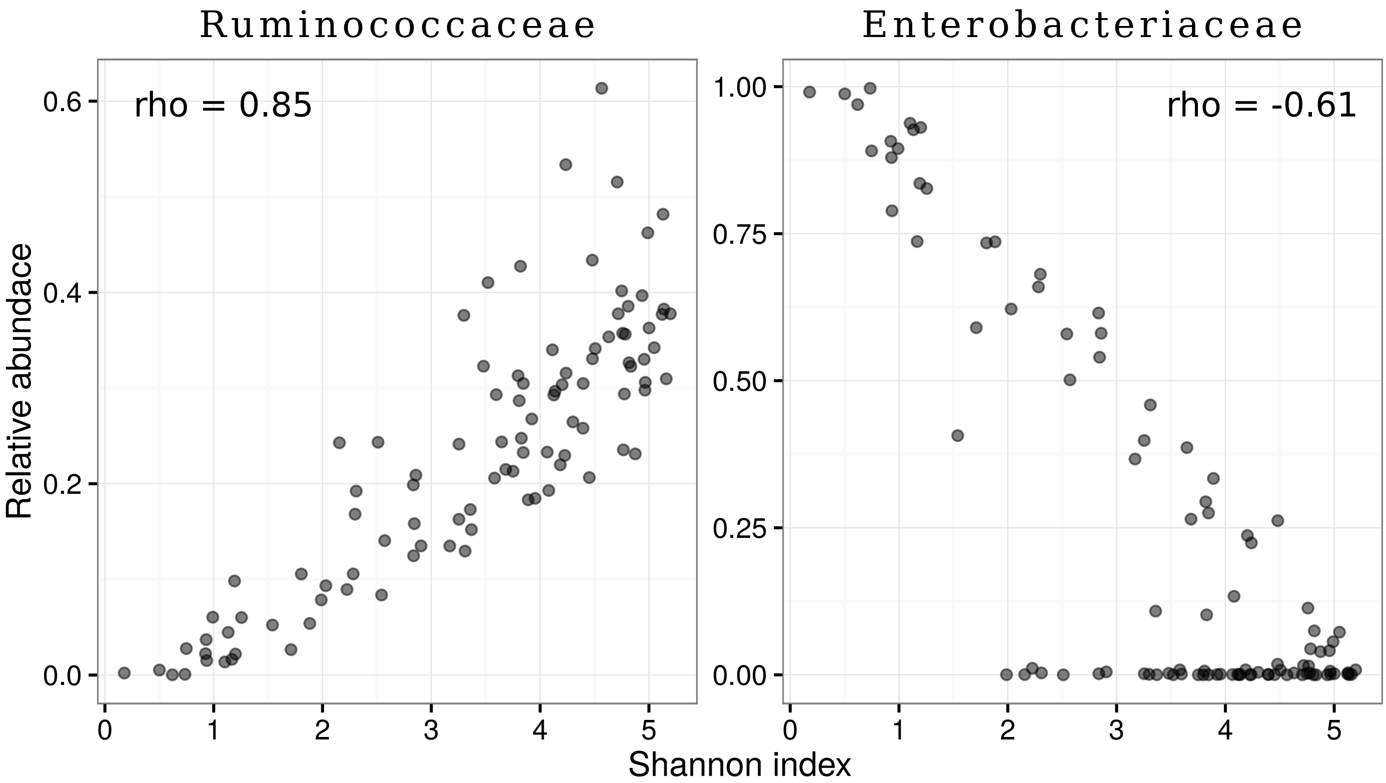

Fig. S2. Comparison of alpha-diversity parameters (richness, Shannon, phylogenetic diversity) between samples of different subspecies for wild and captive individuals. Values were obtained from the mean parameters after 100 rarefied tables. Significant statistical differences were found between wild *O. c. cuniculus* and each of the other category by a pairwise Kruskal-Wallis test with a signification level of 99% ($P < 0.01$).

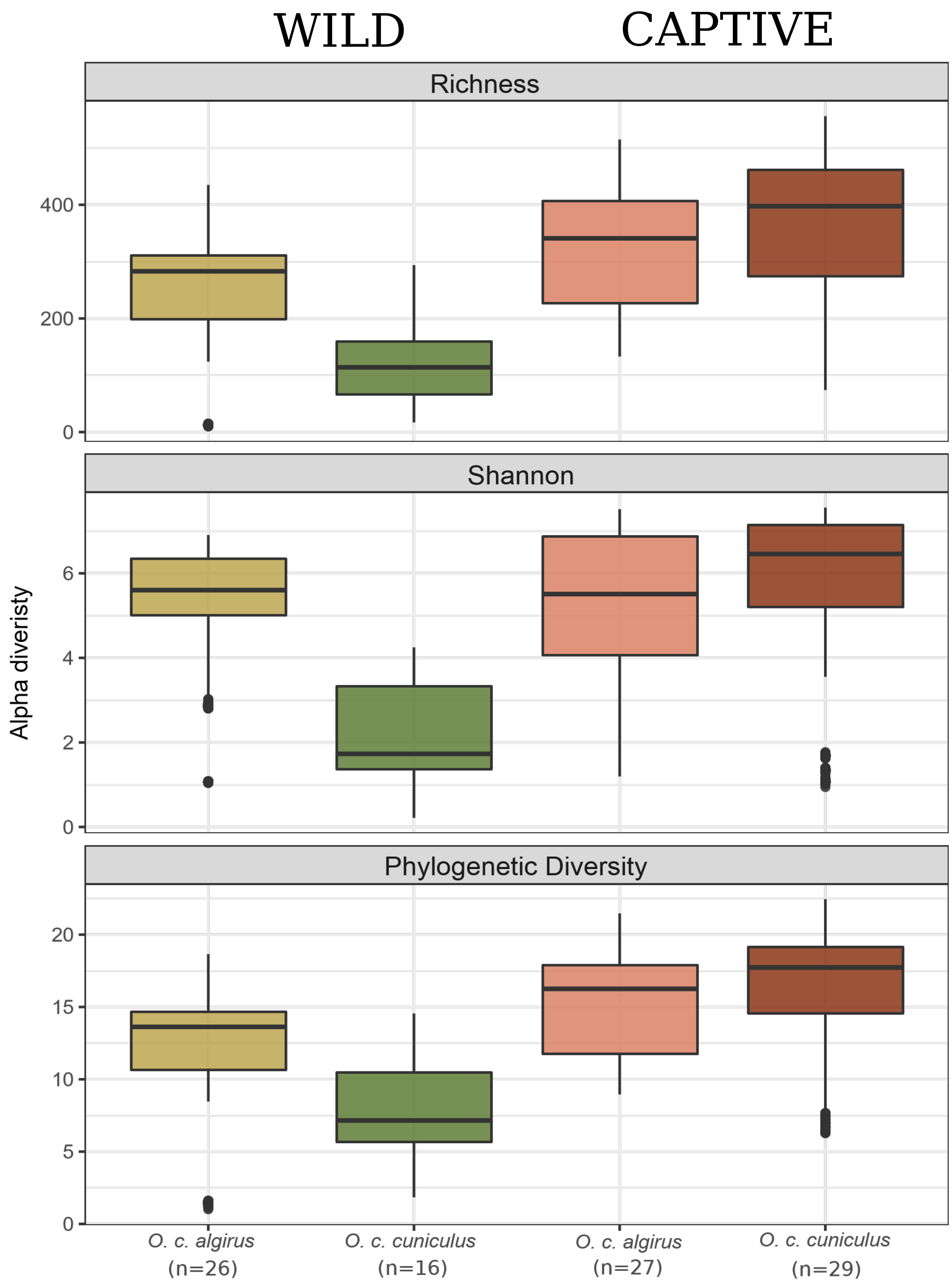

Supplement: Supplementary file 1 — Supplementary Information. [file 41598_2020_80782_MOESM1_ESM.pdf]
